# Opium and risk of gastrointestinal cancer: a case-control study

**DOI:** 10.3906/sag-1907-100

**Published:** 2020-06-23

**Authors:** Reza VAZIRINEJAD, Roya NAJAFIPOUR, Mohsen REZAEIAN, Alireza GHAZIZADEH, Fateme DOUST MOHAMMADI

**Affiliations:** 1 Department of Epidemiology and Biostatics, Medical School, Rafsanjan University of Medical Sciences, Rafsanjan Iran; 2 Department of Public Health, School of Health, Bam University of Medical Sciences, Bam Iran; 3 Department of Epidemiology and Biostatics, Medical School, Rafsanjan University of Medical Sciences, Rafsanjan Iran; 4 Department of Internal Medicine, Medical School, Rafsanjan University of Medical Sciences, Rafsanjan Iran; 5 Department of Epidemiology and Biostatics, Medical School, Rafsanjan University of Medical Sciences, Rafsanjan Iran

**Keywords:** Gastrointestinal cancer, opium, case-control study

## Abstract

**Background/aim:**

Gastrointestinal (GI) cancers are among the most common cancers in the world. Many risk factors may increase the chance of developing GI cancers. In recent years, a number of epidemiological studies have reported evidence of carcinogenic effects of opium in humans. This study aimed to investigate the relationship between opium use and GI cancer.

**Materials and methods:**

This case-control study was performed on 95 patients with GI cancer and 190 healthy individuals (matched for age, sex, place of residence, and smoking) in Rafsanjan, Iran, in 2018. Diet information, as well as history of smoking, the use of hookah, opium, and its derivatives was collected using a checklist in interview sessions. Conditional logistic regression was performed to investigate the proposed relationship and to estimate odds ratios (OR).

**Results:**

After adjusting the confounding variables, the use of opium was significantly associated with an increased risk of GI cancer development (OR = 5.95, 95% CI: 2.4–14.9). Also, a dose-response association was found between the cumulative use of opium and the risk of GI cancers. Consumption of fruit and vegetables reduced the risk of developing GI cancers in opium users (OR = 4.9 and 4.7, respectively).

**Conclusion:**

Opium, in the form used among drug users in this area, can lead to an increased risk of GI cancers. Fruit and vegetables have a protective and modifying effect on the risk of GI cancer development caused by opium consumption.

## 1. Introduction

The use of opioids, including opium and its natural derivatives, is one of the most important global concerns today [1]. According to the United Nations Office on Drugs and Crime (UNODC), about 19.4 million people (0.4%) in the world (15–64 years old) have been opiate users [1]. More than half (58%) of the global opiate users live in Asia. The highest prevalence of opiate use is reported in the Middle East and Southwest Asia (0.9%) [1]. Because of Iran’s long border with Afghanistan, the world’s largest opium producer, opium is easily available in Iran [2], leading to a high level of consumption in this country [3–5]. 

Laboratory research on the carcinogenicity of opium and its mechanism began in the late 1970s [6]. In the recent years, a number of epidemiological studies have also provided evidence of the effects of opium use on human cancers and its role in the development of oral [7], laryngeal [8,9], lung [10,11], bladder [12,13], and gastrointestinal (GI) cancer [14,15]. However, the results obtained in this area are limited and more evidence is necessary to make a causal association between opium use and the risk of developing cancer. 

GI cancers are among the most common cancers in the world [16]. According to the World Health Organization (WHO) in 2018, colorectal and stomach cancer are the third and fourth most common cancers among men. Also, following breast cancer, colorectal cancer is the second commonly occurring cancers among women worldwide 11World Health Organization. Cancer. Available at: http://www.who.int/cancer/en/. Updated 2019. (Last accessed April 2019). GI cancers generally play an important role in the high rates of mortality caused by cancer and related diseases in Asia [17]. Studies have also shown that the burden of GI cancers in Asia is increasing [18]. Evidence has shown that in recent years, GI cancers have been among the most common cancers in Iran, increasing in both incidence and mortality 22 World Health Organization. International Agency for Research on Cancer. Population Fact Sheets. Available at: http://gco.iarc.fr/today/data/factsheets/populations/364-iran-islamic-republic-of-fact-sheets.pdf. Updated 2019. (Last accessed April 2019) [19–22]. In this study, GI cancers include oesophageal, gastric, pancreatic, and colorectal cancers.

Previous studies show that some of the alkaloids in opium may cause digestive system dysfunction. For example, opium consumption activates μ opioid receptors, which are widely distributed throughout the gastrointestinal tract. Activation of these receptors results in decreased peristalsis motility in the gastrointestinal tract [23,24]. As a result, the duration of time in which food passes through the system, increasing the time the system is exposed to the consumed carcinogens. A recent study of Kerman Coronary Artery Disease Risk factors (KERCADRS) showed a 10.6% prevalence of opium use in Kerman adult population aged 15–75 [25]. The present study was performed to investigate the effect of opium on the incidence of GI cancers in Rafsanjan, the third most populous city in Kerman province with a high prevalence rate of GI cancers [26] and opium consumption [4,27].

## 2. Materials and methods

### 2.1. Study design

In the present study, 285 individuals were studied in 2 groups of case and control in 2018. Patients with GI cancers – oesophageal, gastric, pancreatic, and colorectal cancers – and a minimum age of 18 referred to the oncology department of Ali ibn Abi Talib Hospital – the only medical centre for patients diagnosed with cancer in Rafsanjan city – were selected by convenience sampling method and were enrolled after obtaining informed consent. All of these people lived in areas covered by Rafsanjan city, and their cancer had been diagnosed in the previous 2 years (recruiting new cases) based on the results of pathology tests. Non-Iranian people and those who also consumed alcohol, nas, and other opioid drugs such as heroin, methadone, and morphine were excluded. Other exclusion criteria were the history of cancer and the presence of cancer in other organs. 

To determine the sample size, the proportion of opium and its derivatives used in the affected group (p1) and in the healthy group (p2), were obtained from the Shakeri et al. study as 0.4 and 0.2, respectively [28]. Considering the significance level of 0.05 (α = 0.05) and the power of 80% (β = 0.2), using the formula 

 the required sample size was estimated 80 for the case group. Finally, about 20% was added to the sample size to compensate for potential losses. Therefore, 95 patients with GI cancers were enrolled as the case group. 

After selecting cases, 2 matched controls (a total of 190) were selected for each patient, 1 among close relatives and 1 among the neighbours of the patient in order to control the effect of heredity and environment, respectively. After collecting information from each patient, they were asked to report 2 healthy (cancer-free) individuals, 1 of their close relatives and the other among their neighbours. It is noteworthy that those who were introduced were required to be at most 2 years older or younger than the patient as well as having the same sex. Accordingly, for each of the patients who were smokers, the 2 matched controls also had to be cigarette smokers with the same age and sex requirements as those mentioned above. Therefore, the subjects in the case and control groups were individually matched according to the variables such as age (±2 years), sex, place of residence (urban or rural area), and smoking.

The required information was collected using a 3-part checklist in individual interview sessions. Demographic items asking about age, sex, place of residence, level of education, marital status, and employment status formed the first part of the checklist. There were also 2 questions about the year of diagnosis and family history of cancer in patients. In the second part, respondents answered questions about their diet and the consumption of different food groups. The third part of the checklist included questions about the history of using raw opium (crude opium), opium sap (refined opium extract), burned opium (opium dross left in the pipe after smoking opium), cigarettes, and hookah. This part also included questions about details of the use of opium and its derivatives; details included starting date and the duration of consumption, the average daily intake (measured in mesghal, equal to 4.6 g), and usage method (oral or inhaled). The details on cigarette smoking including the age of starting, duration of consumption, and the average amount of smoking per day were also acquired. At the end of this section, there was 1 question to measure the history of indirect exposure to the smoke of opium. 

In order to reduce the reverse causality effect, we recorded the history of cigarette, opium, and opium derivative usage much before the time of cancer diagnosis. In addition, as cancer patients may have used opium to reduce their pain after the beginning of the cancer, the history of the use of opium and its derivatives in people who initiated opium use less than one year before the diagnosis, was not recorded [28]. Diet information related to 10 years before the diagnosis of the case group, was also gathered. As respondents in the control group were individually matched with each case based on age, related information was collected from controls for the same 10-year period as the associated case. Standard interviews were conducted in a relaxed and friendly atmosphere by a trained expert. To reduce potential interviewer bias and interpersonal variability all interviews were conducted by only 1 expert. Similarly, in order to minimize underreporting, questions about opium use and its derivatives were asked at the end of the interview and after gaining the trust of the participants. 

This research project was approved by the Ethics Committee of Rafsanjan University of Medical Sciences. In order to address ethical concerns and to maximize the individuals’ participation, the purpose of the study was explained to each participant before the interview and informed consent was obtained. Also, the participants’ information was kept completely confidential and their names were not mentioned at any stage of the study. 

### 2.2. Data analysis

The cumulative use of opium was calculated by multiplying the average daily use (measured in mesghal, 4.6 g) by the duration of use (year) [29]. Regarding the effective role of red meat, fruit, and vegetables on the incidence of GI cancers, the effect of these variables was initially controlled by stratification method and their role in relation to opium use and GI cancers was assessed. Then, the conditional logistic regression model was used to determine the association of opium use and GI cancers. In the final models, the effects of variables such as education level, family history of cancer, consumption of red meat, fruit, and vegetables were adjusted and the relationship was reported as adjusted odds ratio (OR) and a 95% confidence interval for odds ratio and P-value for assessing the significance of the relationship. The overall goodness of fit of the model was then evaluated using Hosmer-Lemeshow goodness-of-fit test and the area under the ROC (Receiver Operating Characteristic) curve. All data were analysed using SPSS version 21.0 (IBM Corp., Armonk, NY, USA) and SAS version 9.2. The significance level was considered as 0.05.

## 3. Results

The study subjects were 95 patients aged between 22 and 90 with GI cancers, out of whom 8 (8.42%), 41 (43.16%), 7 (7.37%), and 39 (41.05%) were suffering from oesophageal, stomach, pancreatic, and colorectal cancers, respectively. Also, 190 healthy individuals were recruited as the control group. The controls were matched to cases by age, sex, place of residence, and smoking. The mean and standard deviation of age in the case and control groups were 61.54 ± 15.64 and 61.43 ± 15.49 years, respectively (P = 0.975). The participants matched according to sex, place of residence, and smoking, were 55.79% male and 66.32% urbanite, and 26.32% of them were cigarette smokers. Also, there was no significant difference between case and control groups regarding mean cigarettes smoked per day **(**4.02 ± 8.47 and 3.61 ± 6.75 respectively, P = 0.654) and duration of use (7.80 ± 15.58 and 7.50 ± 14.9 years respectively, P = 0.875). Similarly, the 2 groups had no significant difference regarding marital status and history of hookah use. However, the difference between 2 groups in education level and family history of developing cancer was significant. Education level was higher in the control group and the case group also reported a significant family history of cancer among their first-degree family members (Table 1). 

**Table 1:** Frequency distribution of patients with gastrointestinal cancer (case group) and control subjects based on some of the demographic variables.

Variable	Variable levels	Case (n = 95)		Control (n = 190)		* P-value
		Number	Percent	Number	Percent	
Gender	Female	42	44.21	84	44.21	0.999
	Male	53	55.79	106	55.79	
Place of residence	Urban	63	66.32	126	66.32	0.999
	Rural	32	33.68	64	33.68	
Marital status	Single	3	3.16	2	1.05	0.337
	Married	92	96.84	188	98.95	
Cigarette smoking	Yes	25	26.32	50	26.32	0.999
	No	70	73.68	140	73.68	
Hookah use	Yes	1	1.05	3	1.58	0.999
	No	94	98.95	187	98.42	
Level of education	Uneducated	35	36.84	55	28.95	<0.001
	High school	39	41.05	37	19.47	
	Diploma and higher	21	21.11	98	51.58	
Family history ofcancer	Yes	15	15.79	12	6.32	0.010
	No	80	84.21	178	93.68	

*Chi-square test was used if the assumptions were accurate; otherwise, Fisher’s exact test was used.

Table 2 includes the amount and the type of food consumed as well as duration of opium use, daily intake, and cumulative consumption of opium in the case and control groups. The results showed that monthly use of red meat in the control group was higher than it was in the case group. On the other hand, the mean amount of fruit and vegetable consumption per week was higher in the case group. About 26.32% (n = 25) of the cases and 6.32% (n = 12) of the controls used opium (P < 0.001). Opium use was more common among men than women, with men comprising 21 (84%) and 11 (92%) of opium users in the case and control groups, respectively. None of the participants reported consumption of opium dross. Only 3 (1.64%) in the case group consumed refined opium in addition to opium, so separate analyses were not performed on these subjects. Additionally, all opium and refined opium users used inhalation method. As shown in Table 2, the average duration and daily amount of opium consumption in the case group were higher than they were in the control group. As a result, the cumulative use of opium was higher in the case group. 

**Table 2:** The mean frequency of consumption of some food types, duration of opium use, daily consumption of opium, and the cumulative consumption of opium in the case (patients with gastrointestinal cancer) and control groups.

Variable	Case (n = 95)		Control (n = 190)		*P Value
	Mean	Standard Deviation	Mean	Standard Deviation	
Monthly consumption of red meat (times)	9.09	5.21	10.61	4.37	0.010
Weekly fruit consumption (times)	4.65	4.16	2.34	1.56	<0.001
Weekly vegetable consumption (times)	2.68	2.35	2.02	1.47	0.013
Duration of opium use (years)	7.31	13.67	1.61	7.02	<0.001
Daily amount of opium consumption (mesghal**)	0.54	1.15	0.07	0.33	<0.001
Cumulative consumption of opium (mesghal/year)	14.62	30.78	1.93	9.64	<0.001

*Independent t-test, **1 mesghal = 4.608 g.

Our investigation showed that 26.32% (n = 25) of the case group and 2.63% (n = 5) of the controls had indirect exposure to the smoke of opium (P < 0.001). Indirect exposure means constant exposure to the smoke of opium used by a family member or friend. As some individuals, especially in the control group, may have avoided telling the truth in order to preserve friend and family secrets and in order to prevent subsequent information bias, no more analysis was performed on this data. 

To control the effect of confounding variables such as consumption of red meat, fruit, and vegetables on the relationship between opium use and GI cancers, after categorizing individuals based on the consumption of each of these food types (according to the Iranian dietary standards), we used stratification method (Table 3). When stratified by weekly fruit consumption, more association was found in subjects with less fruit consumption (OR = 6.5, 95% CI: 2.8–15.2) than those who consumed more fruit on a weekly basis (OR = 2.1, 95% CI: 0.4–11.1). Also, the risk of GI cancers due to opium use was higher in individuals who consumed less vegetables during the week (OR = 6.4, 95% CI: 2.8–14.9) compared to those who consumed more vegetables (OR = 1.7, 95% CI: 0.3–8.5). The risk of GI cancers due to opium use was higher in those who consumed less red meat (OR = 6.7, 95% CI: 2.3–19.4) than those who consumed more meat every month (OR = 4.2, 95% CI: 1.4–12.9). After adjusting the effect of consumption of fruit, vegetables, and red meat, the risk of developing GI cancers in opium users was about 4.9, 4.7, and 5.5 times higher than it was among those who did not use opium (P < 0.001). Therefore, it may be concluded that the consumption of fruit, vegetables, and red meat can modify the effect of opium use on the incidence of GI cancers as an effect modifier. This means that the risk of the effect of opium consumption on GI cancers decreases with the consumption of more fruit, vegetables, and red meat. 

**Table 3:** The extent of opium use as a risk factor for gastrointestinal cancers after stratification of subjects based on weekly frequency of fruit and vegetable consumption and the monthly rate of red meat consumption.

Amount and type of food consumption	Opium consumption	The Odds ratio for gastrointestinal cancers (95% CI)	*P-value
Weekly consumption of fruit 1–3 times	No Yes	1 6.484 (2.771–15.173)	<0.001
4 times or more	No Yes	1 2.074 (0.387–11.113)	0.474
Total	No Yes	1 4.872 (2.238–10.604)	<0.001
Weekly consumption of vegetables 1–3 times	No Yes	1 6.433 (2.771–14.936)	<0.001
4 times or more	No Yes	1 1.667 (0.328–8.462)	0.693
Total	No Yes	1 4.719 (2.224–9.924)	< 0.001
Monthly consumption of red meat 1–10 times	No Yes	1 6.728 (2.338–19.361)	< 0.001
11 times or More	No Yes	1 4.224 (1.386–12.868)	0.022
Total	No Yes	1 5.540 (2.586–11.870)	< 0.001

* Chi-square test was used for presupposed conditions; otherwise, Fisher’s exact test was used.

Table 4 shows the results of using conditional logistic regression models to evaluate the effect of opium use and cumulative consumption of opium on the incidence of GI cancers. The results showed that after adjusting the effects of variables such as educational level, family history of cancer, and the consumption of red meat, fruit, and vegetables, the effect of the use of opium on GI cancers was significant (OR = 5.95, 95% CI: 2.4–14.9). Also, the results showed that, after fixing the effects of the confounding variables, the increase in cumulative consumption of opium per unit (mesghal per year) increased the risk of cancer by 4.0%, which indicated a dose-response relationship between opium use and the incidence of GI cancers. The result of the Hosmer-Lemeshow test was greater than 0.05, confirming the calibration of the models. On the other hand, the area under the ROC curve was greater than 0.8, demonstrating the excellent ability of the model to distinguish between sick and healthy people. 

**Table 4:** Crude and adjusted odds ratios for the effect of opium use and cumulative opium consumption on the risk of gastrointestinal cancers.

Variable	Crude odds ratio (95% CI)	P-value	*Adjusted odds ratio (95% CI)	P-value	Hosmer-Lemeshow goodness-of-fit test	Area under ROC curve
Opium consumption	5.298 (2.52–11.12)	0.001>	5.954 (2.37–14.99)	0.0002	0.2764	0.8619
Cumulative opium consumption	1.038 (1.02–1.06)	0.001>	1.040 (1.02–1.06)	0.0002	0.8810	0.8628

*Adjusted for education level, family history of cancer, consumption of red meat, fruit and vegetables.

## 4. Discussion

This study aimed to investigate the effect of opium use on the risk of GI cancers and determine the extent of this effect in the development of these cancers. The results of the study showed that opium can increase the risk of GI cancers. Also, the risk of developing these cancers increased with an increase in cumulative consumption of opium (amount and duration). Even after adjusting and controlling the effects of potential confounding variables such as education level, family history of cancer, and consumption of red meat, fruit, and vegetables, the odds ratio was higher than the crude state (adjusted OR = 5.95 compared to crude OR = 5.30), which indicated a strong association between opium use and GI cancers. The results of previous studies have shown that the prevalence of opium use is higher in lower educated people [25]. Therefore, in this study the level of participants’ education was assessed and scored at 3 levels as illiterate (uneducated, not attending school), high school (up to 12 years of schooling), and diploma and higher. Then the effect of this confounder variable was adjusted.

The results of this study are supported by evidence from previous epidemiological and laboratory studies [30]. Previous studies have shown the evidence of an increased risk of oesophageal [29], stomach [28,31], pancreatic [32,33], and colorectal [34] cancers, i.e. both upper and lower gastrointestinal tract cancers [14,15], as well as GI cancer deaths [35] due to opium use. In addition, the results of laboratory studies conducted to investigate the carcinogenic mechanisms of opium have shown that opium components have mutagenic properties and cause mutations in salmonella bacteria [6,36]. Moreover, the pyrolysates derived from the thermal decomposition of opium and its main alkaloid morphine could induce the exchange of genetic information between sister chromatids in human lymphocyte and hamster ovarian cells and also morphological changes in hamster embryonic cells [37,38]. Topical, subcutaneous, intra-tracheal, and intra-gastric administration of these materials in mice also led to cancerous activity in these parts [38]. The effects might also be due to the substances added to opium in the preparation process, for example, the results of studies have shown that the presence of high levels of mineral lead in opium [39] leads to an increase in the level of lead in the blood after its use [40,41], which can cause severe toxicity and even cancer [42–44].

We reported the consumption of opium in 26% of the case and 6% of the control group subjects. The results of similar studies conducted in different parts of the country suggested that opioid use varies between 16%–38% in people with cancer and 5%–18% in controls [14, 15, 28, 29, 32, 34]. The difference in results can be justified considering the difference in sample size, the prevalence of opium uses in different parts of Iran, and also the time of opium consumption (before or after the beginning of cancer). 

One of the most important parts of the present study was the results of the study on the effect of diet on the relationship between the opium use and GI cancers, suggesting that more fruit and vegetables can significantly reduce the risk of GI cancers. Similarly, the results of recent reviews showed that the consumption of fruit and vegetables was associated with a reduction in the risk of gastric and colorectal cancer [44,46]. This part of our results is consistent with the evidence obtained from similar studies conducted in Iran [15,34]. These results could be interpreted as opium use affects digestive system function by increasing the duration of digestive tract exposure to food and consumed carcinogens through reducing the movement of food through the system [24,47]. By increasing the amount of fibre intake, the consumption of fruit and vegetables can reduce this effect of opium consumption on the digestive tract [48].

In contrast to the evidence obtained from previous studies of the role of red meat consumption in the development of GI cancers [49], our results showed that higher red meat consumption reduces the effect of opium consumption on GI cancers. The difference in our results compared to the previous results can be attributed to the fact that due to the low consumption of red meat in the standard Iranian diet, the participants still consumed a relatively lower amount of red meat than participants in the studies in question. In other words, the majority of participants were in reality not eating excessive amounts of red meat at all. 

We designed a strict inclusion and exclusion protocol, and the baseline alignment within groups bolstered the strength of this case-control study. Also, consideration was given to the effective role of variables such as age, sex, environmental and hereditary factors, and smoking in the individual matching process. In addition, using neighbours and relatives (population-based controls) led to the control of environmental, genetic, and socio-economic factors [50], which in turn increased the validity of the results of this study compared to similar studies. Moreover, we studied the effect of consumption of fruit, vegetables, and red meat and the moderating role of these factors on the relationship between opium use and the incidence of GI cancers, which had not been considered in previous studies. 

It may be argued that the patients may have used nonopioid analgesics affecting the outcome of the study. Analgesics are prescribed after the patients have developed cancer; therefore, it may not affect the effect of opium on the occurrence of cancer. Therefore, this will not be a confounding. 

However, this study had some limitations. Firstly, the analgesic usage, other than the opioids, before the cancer development is not evaluated in the study. As opium has analgesic effects, the case group may have used less nonopium analgesics before cancer development. However, the frequency of analgesic usage is low in general population and it does not seem that this difference has been a confounder in the development of GI cancer. Secondly, there is a possibility of recall bias and underreporting of opium use, especially in the control group, which we attempted to minimize this with the use of standard interviewing techniques.

The results of this research showed that there was a strong association between opium use and the risk of GI cancers, proposing a convincing explanation for the high incidence of GI cancers in areas with high prevalence of opium use. However, it is suggested that more studies need to be conducted allowing accurate measurement of the diet and other confounding factors. It is also important to study the effects of other drugs, especially industrial narcotics, on cancer, considering the increasing use of these drugs in the society.

## Acknowledgements

The present research is based on master’s thesis in epidemiology of Rafsanjan University of Medical Sciences. The authors thank the Research Deputy of Rafsanjan University of Medical Sciences and Ali ibn Abi Talib Hospital staff for their valuable helps. They sincerely acknowledge the help of Ms. Khalili at Health Deputy Department of Rafsanjan University of Medical Science for her sincere cooperation. They also thank the study participants for their cooperation. The authors would like to acknowledge the support from Research Deputy of Rafsanjan University of Medical Sciences. 
